# Toll like Receptor 3 & 4 Responses of Human Turbinate Derived Mesenchymal Stem Cells: Stimulation by Double Stranded RNA and Lipopolysaccharide

**DOI:** 10.1371/journal.pone.0101558

**Published:** 2014-07-08

**Authors:** Se Hwan Hwang, Hye Kyung Cho, Sang Hi Park, WeonSun Lee, Hee Jin Lee, Dong Chang Lee, Jeong Hoon Oh, Sun Hwa Park, Tai-Gyu Kim, Hyun-Jung Sohn, Jun Myung Kang, Sung Won Kim

**Affiliations:** 1 Department of Otolaryngology-Head and Neck Surgery, The Catholic University of Korea, College of Medicine, Seoul, Korea; 2 Department of Pediatrics, Graduate School of Medicine, Gachon University, Incheon, Korea; 3 Institute of Clinical Medicine Research, College of Medicine, Catholic University of Korea, Seoul, Korea; 4 Department of Biomedical Science, The Catholic University of Korea, College of Medicine, Seoul, Korea; 5 Catholic Hematopoietic Stem Cell Bank, College of Medicine, The Catholic University of Korea, Seoul, Korea; 6 Department of Microbiology and Immunology, College of Medicine, The Catholic University of Korea, Seoul, Korea; University of London, St George's, United Kingdom

## Abstract

**Background and objectives:**

Multipotent mesenchymal stromal cells (MSCs) represent a promising cell-based therapy for a number of inflammatory or autoimmune diseases. Herein, Toll like receptor (TLR) expression by MSCs and their immune regulatory roles are investigated. In this study, we investigated the influence of TLR on the immune response, proliferation, and differentiation potential of human turbinated MSC (hTMSC) cultures *in vitro*.

**Subjects and Methods:**

After isolating hTMSCs from discarded inferior turbinate tissue, FACS analysis was used to assess the expression of TLRs such as TLR2, TLR3, TLR4, and TLR5 in hTMSCs and cell proliferation was assessed using a cell counting kit (CCK)-8. Cytokine and chemokine secretions were analyzed with multiplex immunoassays for IL-1α, IL-1β, IL-4, IL-6, IL-8, IL-10, IL-12, IP-10 (CXCL10), RANTES (CCL5), TNF-a, GM-CSF, and IFN-γ. The differentiation potential of hTMSCs was evaluated in the osteogenic, chondogenic, and adipogeinc media and analyzed by histology and gene expression related to differentiation.

**Results:**

FACS analysis revealed that TLR3 and TLR4 expression consisted of a relatively high percentage of the surface proteins expressed by hTMSCs. The proliferation of hTMSCs was influenced and significantly increased by the presence of TLR4 agonists. In particular, hTMSCs produced a set of cytokines and chemokines and the expression of IL-6, IL-8, IL-12, IP-10 (CXCL10), RANTES (CCL5), TNF-α, and GM-CSF were up-regulated in response to the TLR4 agonist LPS. The osteogenic and adipogeinc differentiation potential of hTMSCs was not affected by TLR agonists.

**Conclusions:**

We conclude that TLR4 stimulation affects TLR expression, proliferation, and the immunomodulation potential of hTMSCs. Understanding the mechanism behind TLR's influence on hTMSCs and their immunomodulating properties would be useful for providing a novel target to exploit in the improvement of stem cell-based therapeutic strategies.

## Introduction

Members of the family of pattern recognition receptors, Toll like receptors (TLRs) are innate immune receptors. They are expressed on the surfaces of monocytes/macrophages, neutrophils, dendritic cells and endothelial cells; and mediate the activation process of innate immunity cells by recognizing pathogen associated molecular patterns (PAMPs), such as lipopolysaccharides. Activation of TLRs promote the secretion of various inflammatory cytokines such as tumour necrosis factor-α (TNF-α) to induce the expression of costimulatory molecules and initiate adaptive immune responses. Hence, they play a key role in the connection between innate and adaptive immunity [1].

Mesenchymal stromal cells (MSCs) have immunomodulating properties and can inhibit the function of immune cells. These immunologic characteristics make MSCs an interesting tool for cellular therapy. This is supported by a number of studies in experimental models of inflammatory diseases demonstrating an efficient protection against allograft rejection, graft-versus-host disease, experimental autoimmune encephalomyelitis, collagen-induced arthritis, sepsis, and autoimmune myocarditis [2]. Although the specific molecular and cellular mechanisms involved in the immunoregulatory activity of MSCs are still under investigation and remain poorly understood, the discovery of TLRs expression by MSCs recently prompted scientists and clinicians to investigate the potential link between TLR signaling and MSC-mediated immunoregulatory functions [3].

Various tissues have been found to contain MSC-like populations that meet the criteria established to describe bone marrow-derived MSCs (BM-MSCs). However, variations in morphology, growth rates, proliferation potential and differentiation capacity have been reported in various tissue specific MSC-like populations [4]. The immunomodulatory properties of MSCs from different organs have been investigated much, and Chen et al suggested that the MSC niche is unique in each tissue, which can contribute to functional differences [5]. Recently, Raicevic et al. reported that, according to the source from which they are derived, human MSC displayed disparities affecting their functional properties. After activation by inflammation or TLR (poly(I:C) 30 µg/ml and LPS 10 µg/ml), the three MSC types investigated; bone marrow, Wharton's jelly, and adipose derived MSC, differed in TLR expression as well as in the transcription or secretion of several cytokines tested including IL-1β, IL-6, IL-12, IL-27, IL-23, IL-8, CCL5, and IL-1Ra [6]. Therefore, it would be essential to understand the immunomodulatory behaviors of MSCs derived from different origins [5].

The mucosal surfaces of respiratory tracts are continuously exposed to enormous amounts of antigens. The expression of active immune responses against pathogens can frequently result in tissue inflammation and damage. However, the mucosal immune system can discriminate between antigens requiring active immune responses and those requiring tolerance and balance the pro-inflammatory responses with anti-inflammatory responses through active control of immune reponses [7], contributing to the different immunological characteristics of MSC from respiratory mucosa. Understanding the immunomodulatory behavior of MSCs derived from human turbinate tissue (hTMSCs) is therefore necessary. In our study, we aimed to demonstrate that hTMSCs express two analogues of TLRs (TLR3 and TLR4), and that their proliferation, differentiation, and secretion of immune modulating factors are drastically affected by specific TLR-agonist engagement. In particular, we noted diverse responses of the hTMSCs following stimulation of TLR3 and TLR4 by low-level and short-term TLR-priming protocols, respectively [8].

## Materials and Methods

This study was conducted in compliance with the Institutional Review Board of the Catholic Medical Center Clinical Research Coordinating Center (HC13TISI0038), informed consent regulations, and the Declaration of Helsinki. All patients provided informed consent before surgery, and the Institutional Review Board of the Catholic Medical Center Clinical Research Coordinating Center approved all procedures. Participants provide their written informed consent to participate in this study. We obtained informed content from participants themselves.

### Donors

Inferior turbinate tissues were discarded from 5 patients undergoing partial turbinectomy. Patients with antrochoanal polyps, nasal polyposis, congenital immunologic problems, history of systemic or topical medications like steroids and immunosuppressants, hypertension, and prior history of allergic rhinitis, or asthma were excluded.

### Cell isolation

The same amount (0.0366 gram) of inferior turbinate tissues was obtained from tissue discarded during surgery and washed three to five times with saline solution containing gentamicin (Kukje Pharmaceutical Industries, Sungnam, Korea). To isolate hTMSCs, the inferior turbinate tissue was washed at room temperature three times with antibiotic-antimycotic solution (Gibco, Gaithersburg, MD) and twice with phosphate buffered saline (PBS), then cut into 1-mm^3^ pieces. The pieces were placed in a culture dish, and the dish was covered with a sterilized glass cover slide. DMEM (Dulbecco's Modified Eagle Media, Gibco) containing 10% fetal bovine serum (FBS) was added, and the tissues were incubated at 37°C in a 5% CO_2_ atmosphere. The culture medium was changed every two or three days. After 3 weeks of culture, the glass cover slide was removed, and tissues floating in the culture medium were removed by washing. The hTMSCs attached to the bottom of the culture dish were detached using 3 ml of 0.25% trypsin in 1 mM EDTA and the cells were counted with ADAM automated cell counter (Digital Bio, Seoul, Korea). hTMSCs cells in four passages were examined for TLR-agonist stimulation-related changes in immunophenotypical characterization, proliferation, and multipotent differentiation.

### TLR priming protocol

LPS (10 ng/mL, Sigma-Aldrich, St. Louis, MO) and poly(I:C) (1 µg/mL, Sigma-Aldrich) were used as the agonists for TLR4 and TLR3, respectively, as previously described [8]. Following the standard procedure, hMSCs were grown to 60–70% confluency in growth medium (DMEM containing 10% FBS) prior to the start of an experiment. TLR-agonists were added to fresh growth medium and incubated with the cells for 1 hr. The cells were then washed twice in growth medium without the TLR-agonists and assayed as described for the experiments, which mimic the gradient of the danger signals that endogenous hTMSCs encounter and respond to at a distance from the site of injury.

### Immunoassay for cytokines

hTMSCs were plated at a density of 1×10^5^ in 24-well plates, allowed to adhere overnight, then pre-treated with TLR agonists (LPS for TLR4 or poly(I:C) for TLR3) for 1 hr as indicated. Conditioned medium was collected after 48 hr and analyzed with MILLIPLEX MAP human cytokine/chemokine multiplex immunoassay (Millipore, Billerica, MA) for IL-1α, IL-1β, IL-4, IL-6, IL-8, IL-10, IL-12, IP-10 (CXCL10), RANTES (CCL5), TNF-a, GM-CSF, and IFN-γ following the manufacturer's instructions. These experiments were performed at least three times on three individual MSC donor pools.

### Proliferation assay

To generate growth curves, hTMSCs were pre-treated with TLR3 and TLR4 agonists for 1 hr and plated simultaneously into 96-well tissue culture plates at a density of 1500 cells per well. The medium was replaced every 2 days. Cell proliferation was assessed using a cell counting kit (CCK)-8 (Dojindo Laboratories, Kumamoto, Japan), following the manufacturer's instructions. The culture medium was removed and 100 µL of fresh medium containing 10-µL CCK-8 was added to each well. The cells were then incubated at 37°C for 4 h. Cell viability was monitored for 7 days. The optical density values were determined in triplicates at minimum against a reagent blank at a test wavelength of 490 nm and a reference wavelength of 630 nm.

### Characterization by the analysis of cell surface markers on hTMSCs

The specific surface antigens of hTMSCs were characterized by flow cytometry analysis. The cells were incubated into a test tube (BD, Franklin Lakes, NJ) at a density of 1×10^5^ cells/ml, then washed three times with wash buffer (PBS and 3% FBS). The cells were incubated with primary antibody for 40 min with saturating concentrations of monoclonal antibodies CD14 (all anti-human CD from BD Biosciences, San Jose, CA), CD19, CD29, CD34, CD73, CD90, HLA-DR, TLR2 (all anti-human TLR from Abcam, Cambridge, ab9100), TLR3 (ab12085), TLR4 (ab30667), and TLR5 (ab13875). After the cells were washed three times in buffer and centrifuged at 1200 rpm for five minutes, they were resuspended in ice cold PBS and incubated with the secondary antibody for 30 min in the dark at 40°C. Cell fluorescence was evaluated by flow cytometry in a FACS Caliber instrument (BD) and the data were analyzed using Cell Quest software (BD).

### Multilinaege differentiation potential of hTMSCs

To induce osteogenic differentiation, hTMSCs were seeded in 12-well tissue culture plates (2×10^4^ cells/well) and incubated in low-glucose DMEM supplemented with 10% FBS, 100 U/ml penicillin, 100 µg/ml streptomycin, 0.1 µM dexamethasone, 50 µM ascorbate-2-phosphate, and 10 mM β-glycerophosphate (all from Sigma). The induction culture was maintained for 4 weeks, with the medium being replaced every 3 days. The cells were analyzed histologically with alkaline phosphatase (ALP) stain and by RT-PCR.

To induce chondrogenesis in hTMSCs, we created two-dimensional cultures in 24-well plates by seeding cells (2×10^6^/ml). Chondrogenic supplements TGF-β1 (10 ng/ml; Sigma, St. Louis, MO) and IGF-1 (10 ng/ml; Sigma) were added to DMEM supplemented with 10% FBS, 100 nM dexamethasone, 50 µM ascorbic acid-2 phosphate (Sigma), 50 mg/ml insulin-transferrin sodium selenite, 1 mM sodium pyruvate (Gibco), 40 µM proline, and 2 mM L-glutamine. The medium was replaced every 2–3 days. At the end of the second week of culture, the structures were collected and characterized by histological analysis (toluidine blue stain) and RT-PCR.

To induce adipogenic differentiation, hTMSCs were seeded in 6-well plates (3×10^3^ cells/well), maintained for 3 days in α-MEM (Gibco) supplemented with 10% FBS and antibiotics, and then incubated in α-MEM supplemented with 10 µg/ml insulin, 200 µM indomethacin, 0.1 µM dexamethasone, 0.5 µM 3-isobutyl-1-methylxanthine (all from Sigma), and 10% FBS for 21 days. The cells were stained with Oil Red O and observed under an inverted microscope. The cells were analyzed by RT-PCR.

### RNA extraction and RT-PCR of hTMSCs

RNA was collected with RNeasy Mini Kit (QIAGEN, Valencia, CA, USA) from the cells cultured for chondrogenic, osteogenic, adipogenic differentiation. Treatment with deoxyribonuclease 1 (QIAGEN) eliminated genomic DNA, and 1-µg purified RNA was reverse transcribed into first-strand complementary DNA using an iScript reverse transcription kit (Bio-Rad Laboratories, Hercules, CA, USA), which includes a genomic DNA elimination step (QIAGEN). Real-time polymerase chain reaction (RT-PCR) amplification and relative quantification of bone morphogenetic protein-2 (BMP-2), type I collagen, Runt-related transcription factor 2 (Runx2), type II collagen, aggrecan, peroxisome proliferator activated receptor r (PPARr), and AcylCoA synthetase (ACS) were performed using TaqMan gene expression assays (Table 1) (Applied Biosystems, Foster City, CA, USA) on a lightcycler 480 PCR system (Roche, Mannheim, Germany). All assays used similar amplification efficiency, and a delta cycle threshold experimental design was used for relative quantification. The reactions were performed in triplicates in a 20-µL volume using TaqMan probe Master Mix (Roche), and 10-ng complementary DNA was used in each reaction. Glyceraldehyde 3-phosphate dehydrogenase served as an endogenous control. Results were analyzed using lightcycler 480 instrument software 1.2 (Roche).

**Table 1:** Gene expression assays used for real-time polymerase chain reaction for Multilineage differentiation.

Gene	Abbreviation	Reference Sequence	Assay number
bone morphogenetic protein 2	BMP-2	NM_001200	Hs00154192_m1
type I collagen	COL1	NM_000088	Hs00164004_m1
runt-related transcription factor 2	Runx2	NM_001015051	Hs00231692_m1
type II collagen	COL2	NM_001844	Hs00264051_m1
Aggrecan	ACAN	NM_001135	Hs00153936_m1
peroxisome proliferator-activated	PPARγ	NM_138712	Hs01115513_m1
receptor gamma			
acyl-CoA synthetase short-chain family	ACS	NM_001076552	Hs00218766_m1
member 2			
Glyceraldehyde 3-phosphate	GAPDH	NM_002046	Hs99999905_m1

### Allogeneic transwell co-culture of hTMSCs and immune cells

Human peripheral blood mononuclear cells (PBMCs) were obtained from peripheral blood by standard Ficoll-Paque Plus (Amersham Biosciences, Piscataway, NJ, USA) separation. For co-culture experiments using transwell supports, preventing direct cell interaction, the hTMSCs (2×10^4^/well) were seeded in a complete growth medium in 12-well culture plates and were allowed to attach overnight in the presence or absence of poly(I:C) or LPS. Allogeneic PBMCs were added to the upper transwell chamber (12 mm diameter) with a 0.4 mm pore size membrane (transwell chamber, Costar, Cambridge, MA) on the following day at a 10∶1 ratio, and cells were co-cultured for 3 days. At the end of the incubation, PBMCs were harvested by careful pipetting, ultracentrifuged at 600×g for 5 minutes, and finally resuspended in PBS for cell counting. Full integrity of MSC layers was checked in all cases. In addition, cell growth inhibition during 3 days of the incubation was assessed using a cell counting kit (CCK)-8.

### Fluorescence Immunocytochemical Analysis

Intracellular antibody staining was achieved after fixation and permeabilization of the cells as indicated by the manufacturer (cytofix/cytoperm buffers; BD Biosciences, San Jose, CA). Nonspecific antibody reactions were blocked with 0.5% normal goat serum for 1 h at room temperature (RT). As a control, the primary antibody was omitted from staining procedure. Fluorescence immunocytochemistry was performed on cells grown to near confluence (70%) on chamber slides, fixed, and permeabilized with BD cytofix/cytoperm buffer (BD Pharmingen, San Diego,USA). The primary antibodies diluted in stain buffer in the appropriate concentration (ratio of 0.5 ug of antibodies per 2×10^4^ cells) were added for overnigh at room temperature. Next, unbound primary antibody was washed twice with PBS. The secondary antibody (Alexa 488-conjugated anti-IgG; Abcam, Cambridge, UK,) was diluted in stain buffer in the appropriate concentrations and added for 1 hour in the dark. Slides were again washed twice prior to 4,6-diamidino-2-phenylindole(DAPI; Sigma-Aldrich) staining and mounting with antifade mounting medium (Gel Mount; Biomedia Corp., Foster City, CA). Micrographs were taken on a fluorescence microscope (BX50; Olympus, Tokyo, Japan) with IMT iSolution image analysis software.

### Statistical analysis

Statistical analyses were conducted using ‘R’ statistical software (R Foundation for Statistical Computing, Vienna, Austria). The statistical significance of the differences between groups was determined by one-way analysis of variance (ANOVA). A *p*-value <0.05 was considered to indicate significance.

## Results

### Characterization and pattern of TLR expression of hTMSCs by flow cytometry

**Figure 1:**
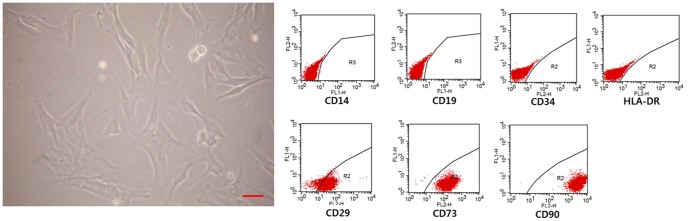
Isolation (A) and fluorescence-activated cell sorting analysis (B) of human turbinate mesenchymal stromal cells (hTMSCs). Adherent cells with fibroblastic morphology were analyzed. Flow cytometry analysis after three passages was used to confirm hTMSCs as positive for CD29, CD73, and CD90, with negative for CD14, CD19, CD34, and HLA-DR. (A) Magnification: ×400. Scale bars: 20 µm.

Adherent cells formed a monolayer of homogenous bipolar spindle-like cells in two to three days. Cell surface markers of in vitro-cultured hTMSCs were examined by flow cytometric analysis. hTMSCs were negative for hematopoietic lineage markers (CD14, CD19, CD34, or HLA-DR), but expressed MSC markers (CD29, CD73, and CD90) (Figure 1).

**Figure 2:**
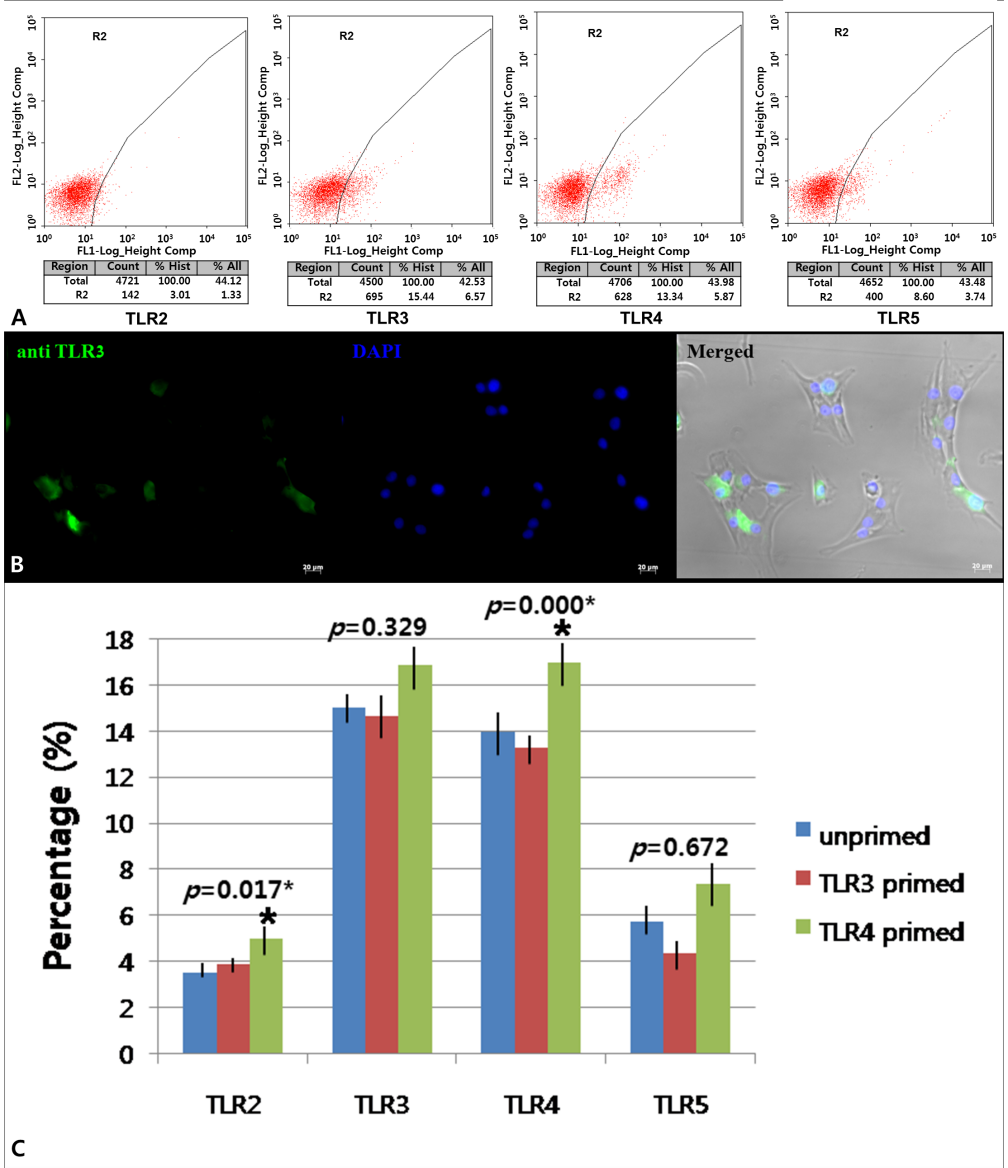
Toll like receptor (TLR) expression (A), fluorescence Immunocytochemical staining (B) for TLR3, and change of TLR expression (C) of hTMSCs. (A): Protein expression was confirmed by flow cytometry analysis as positive for TLR3 (15.03%) and TLR4 (13.94%), with low positivity for TLR2 (3.53%) and TLR5 (5.75%). (B) Antibody staining of TLRs was performed following fixation and membrane permeabilization of the hTMSCs seeded on chamber slides. TLR3 expression was evaluated on hTMSCs by fluorescence microscope. TLR3 was stained diffusely in the cytoplasm (green) and nuclei were counterstained with DAPI (blue). Magnification: ×400. Scale bars: 20 µm. (C): When the cells were cultured in the absence (medium, unprimed) or presence of poly(I:C) (TLR3 primed), or LPS (TLR4 primed), the TLR4 agonist LPS significantly affects the expression of TLR2 and TLR4.

To investigate the expression of TLRs in expanded cells, the protein expression of TLR2, TLR3, TLR4, and TLR5 on hTMSCs were assessed by flow cytometry. As shown in Figure 2A, TLR3 and TLR4 expression comprised a relatively high percentage of the surface proteins expressed by hTMSCs. In contrast, low levels of TLR2 and TLR5 expression were detected in hTMSCs. Moreover, intracytoplasmatic localization of TLR3 was also assessed by fluorescence immunocytochemical stain (Fig. 2 B, C).

Exposure to TLR agonists (LPS for TLR4 or poly(I:C) for TLR3) had no significant effect on the expression of TLR3 and TLR5, as measured by flow cytometry. In contrast, LPS for TLR4 significantly up-regulated the expression of both TLR2 and TLR4. Overall, these results indicate that single treatments with TLR4 agonist result in up-regulation of the expression of TLR2 and TLR4 in hTMSCs (Figure 2B).

### Cytokine and chemokine secretion patterns following the exposure to TLR agonist and allogeneic transwell co-culture

**Figure 3:**
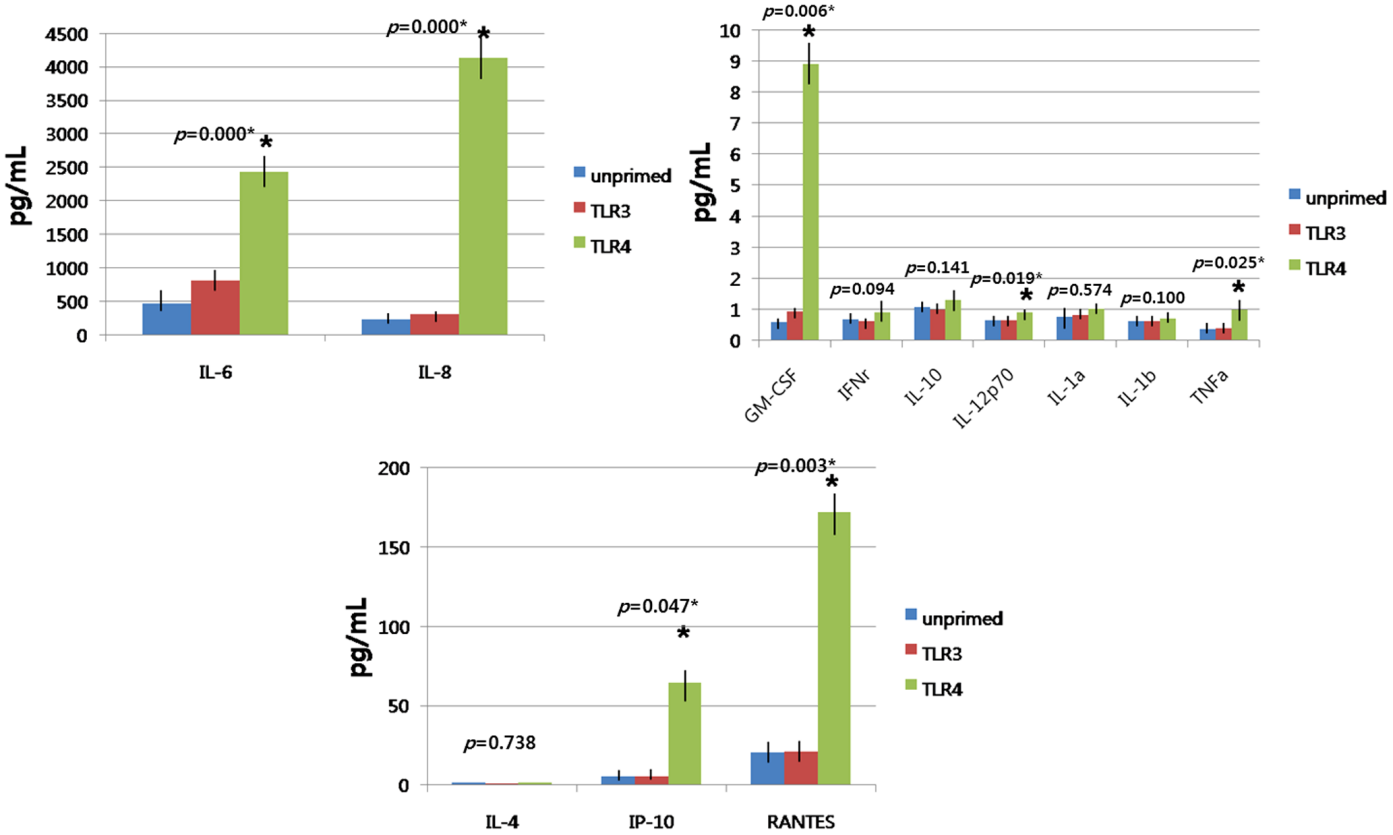
Effects of TLR3 and TLR4 on cytokine and chemokine secretion by hTMSCs. Secretion of cytokines and chemokines such as IL-1α, IL-1β, IL-4, IL-6, IL-8, IL-10, IL-12, IP-10 (CXCL10), RANTES (CCL5), TNF-α, GM-CSF, and IFN-γ of hTMSCs after stimulation with TLR3 and TLR4 were measured with enzyme-linked immunosorbent assay. Among them, IL-4, IL-6, IL-8, IL-10, IP-10 (CXCL10), and RANTES (CCL5) were detectable with mean values of more than 1 ng/ml under standard culture conditions. LPS strongly induced expression of IL-6, IL-8, IL-12, IP-10 (CXCL10), RANTES (CCL5), TNF-α, and GM-CSF, while poly(I:C) did not affect the secretion of cytokines and chemokines.

**Figure 4:**
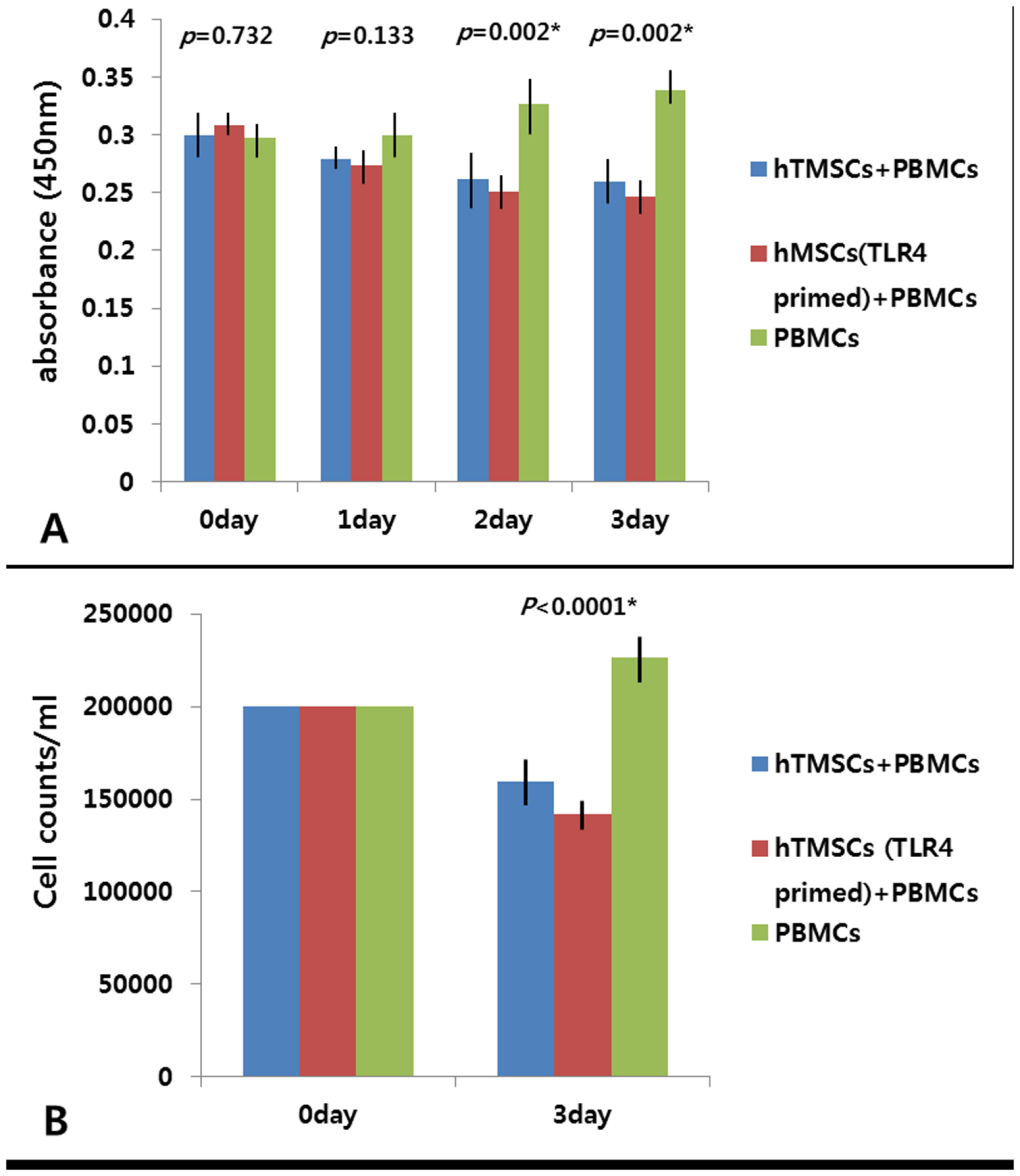
Effects of growth suppressive effect of hTMSCs on human peripheral blood mononuclear cells (PBMC). (A) During hTMSC/PBMC coculture, the proliferation of cocultured PBMCs were suppressed significantly on day 2 and 3, while single-cultured PBMCs showed steady expansion for a period of 3days. (B) The cell counts of PBMCs on day 3 were similar with the pattern of growth inhibition analysis.

We measured a number of cytokines and chemokines known to be involved in immunomodulation, including IL-1α, IL-1β, IL-4, IL-6, IL-8, IL-10, IL-12, IP-10 (CXCL10), RANTES (CCL5), TNF-α, GM-CSF, and IFN-γ. Among them, IL-4, IL-6, IL-8, IL-10, IP-10 (CXCL10), and RANTES (CCL5) were detectable with mean values of more than 1 pg/ml in supernatants harvested from hTMSCs under standard culture conditions. To investigate hTMSCs responsiveness to the exposure of TLR agonists, we next exposed the cells to LPS or poly(I:C). LPS strongly induced expression of IL-6, IL-8, IL-12, IP-10 (CXCL10), RANTES (CCL5), TNF-α, and GM-CSF, unlike poly(I:C) which did not induce expression of these cytokines. Specifically, an approximately 5 to 18-fold increase in IL-6, IL-8, IP-10 (CXCL10), RANTES (CCL5), and GM-CSF was observed with the exposure of TLR4 agonists. IL-12 and TNF-α expression increased by 1.3 to 3-fold, respectively (Figure 3). In addition, to demonstrate that hTMSC are immunologically active, MSC/PBMC coculture experiments were performed. Growth inhibition analysis in PBMCs during 3 days of the incubation showed that these cells in co-culture was significantly decreased from day 2 and was prolonged over time compared with single-cultured PBMCs which expanded slowly (Figure 4A). The measured number of cells at the 3rd day was also similar with the pattern of growth inhibition analysis (Figure 4B). The pretreatment of hTMSC with LPS slightly enhanced the inhibitory effect of hTMSCs on PBMCs. However, there was no statistically significance in these measurements. Overall, these results suggest that hTMSCs are immunologically active and responsive to TLR4 agonist and that TLR4 agonist result in the up-regulation of IL-6, IL-8, IL-12, IP-10 (CXCL10), RANTES (CCL5), TNF-α, and GM-CSF expression in hTMSCs.

### Proliferation assay

**Figure 5:**
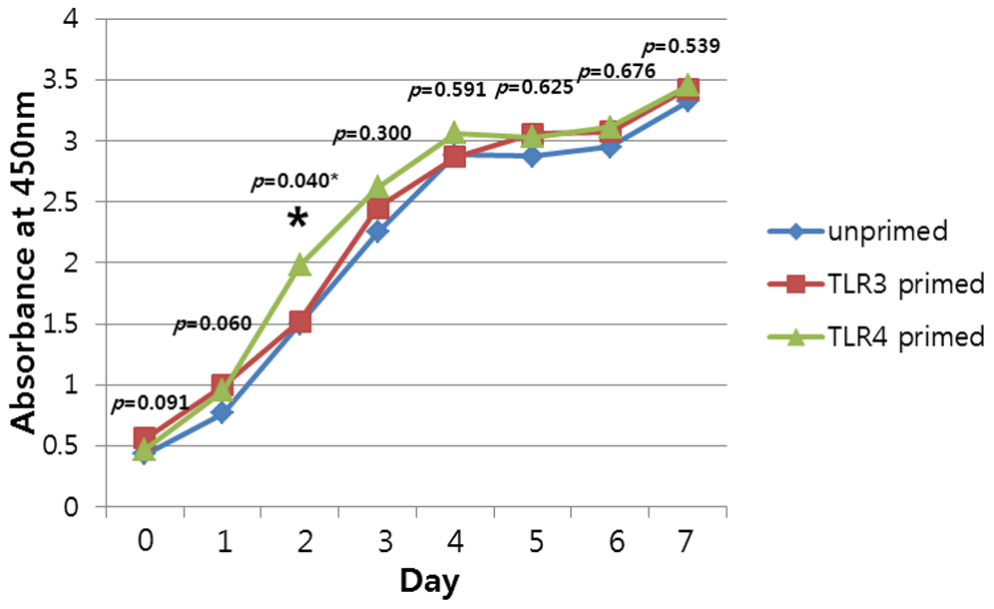
TLR-related changes in cellular proliferation of hTMSCs. Cellular proliferation assay was performed for a period of 7 days. hTMSCs from all groups exhibit rapid proliferation from day 2 to 4, with the hTMSCs exposed to the TLR4 agonist expanding more rapidly compared to the others.

To examine whether TLR activation influences the proliferation of hTMSCs, cell proliferation was monitored over a period of 7 days. All hTMSCs were in a stationary phase during the first day. From days 2 to 4, the cells underwent logarithmic growth, during which hTMSCs from cells exposed to the TLR4 agonist expanded more rapidly than those in the other groups (Figure 5). There were statistically significant differences in proliferation rate among the groups. All cells next entered a lag phase and showed a pattern of confluencing cell numbers during the following days. These results showed that TLR4 agonists influenced the proliferation of hTMSCs.

### Multilineage differentiation potential

Given the reported differences on the effect of TLR3 and TLR4 activation on the tri-lineage (cartilage, bone, fat) differentiation capabilities of hTMSCs, we next measured these differences using reduced amounts of TLR ligand. The hTMSCs were simultaneously induced to differentiate in the presence of TLR3 (1 mg/mL poly(I:C)) and TLR4 agonists (10 ng/mL LPS) for the duration of the differentiation assays in the inductive medium for 2 weeks.

**Figure 6:**
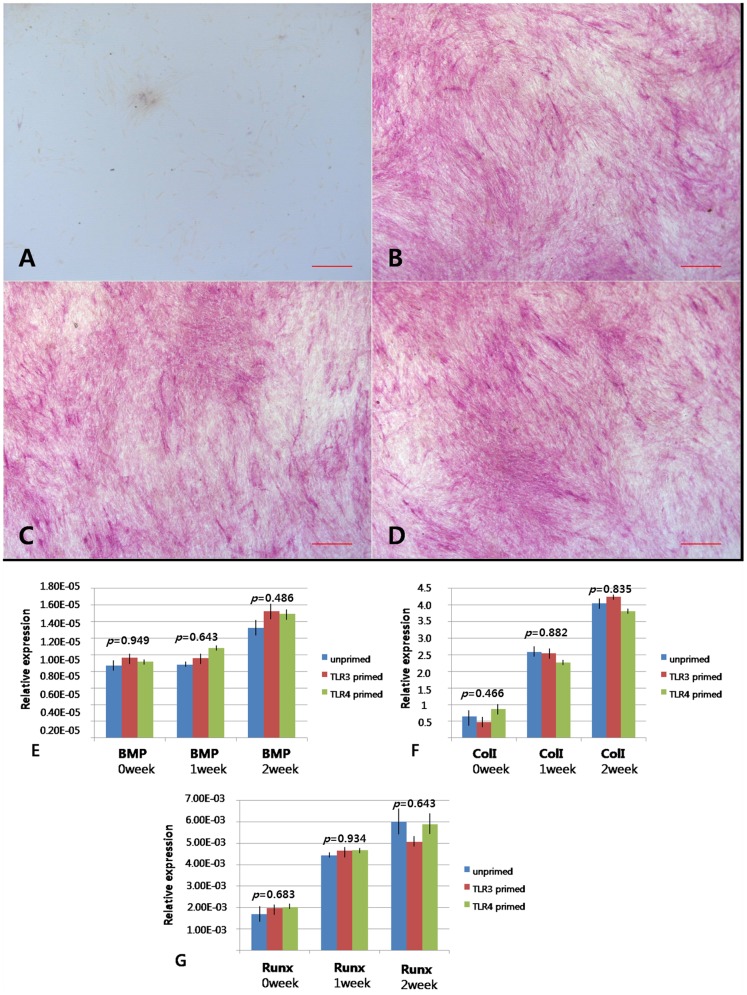
TLR-related change in osteogenic differentiation potential of hTMSCs. Cells were cultured in osteogenic induction medium (A). Cells aggregated, formed nodules, and accumulated calcium deposits over the 2-week period (B–D). B–D: Alkaline phosphatase staining on cultured hTMSCs for all groups. (B: unprimed, C: TLR3 primed, D: TLR4 primed). Scale bars (A–D): 20 µm. No differences in visible color density were observed among the three groups. The mRNA expression of bone morphogenetic protein-2 (E), type I collagen (F), Runt-related transcription factor 2 (G), and in hTMSCs induced to undergo osteogenic differentiation was detected by RT-PCR. The expression levels of osteoblast-specific genes did not differ among the groups.

Cells exposed to osteogenic medium showed direct evidence of calcium mineralization, as determined by ALP staining which could visualize the alkaline phosphatase activity as a red stain. The data from a visual assessment demonstrated that hTMSCs from all groups showed similar amounts of alkaline phosphatase staining (Figures 6A, 6B, 6C and 6D). Analysis by RT-PCR revealed the expression of mRNAs encoding BMP-2, type I collagen, and Runx2 in these cells. The expression levels of osteoblast-specific genes did not differ among the three groups (Figure. 6E–G). On the basis of the result that hTMSCs showed consistently strong and increasing expression of osteogenic differentiation markers regardless of exposure to TLR agonists and the expression levels of osteoblast-specific genes did not differ among the groups, we concluded that osteogenic differentiation of hTMSCs was not affected by TLRs.

**Figure 7:**
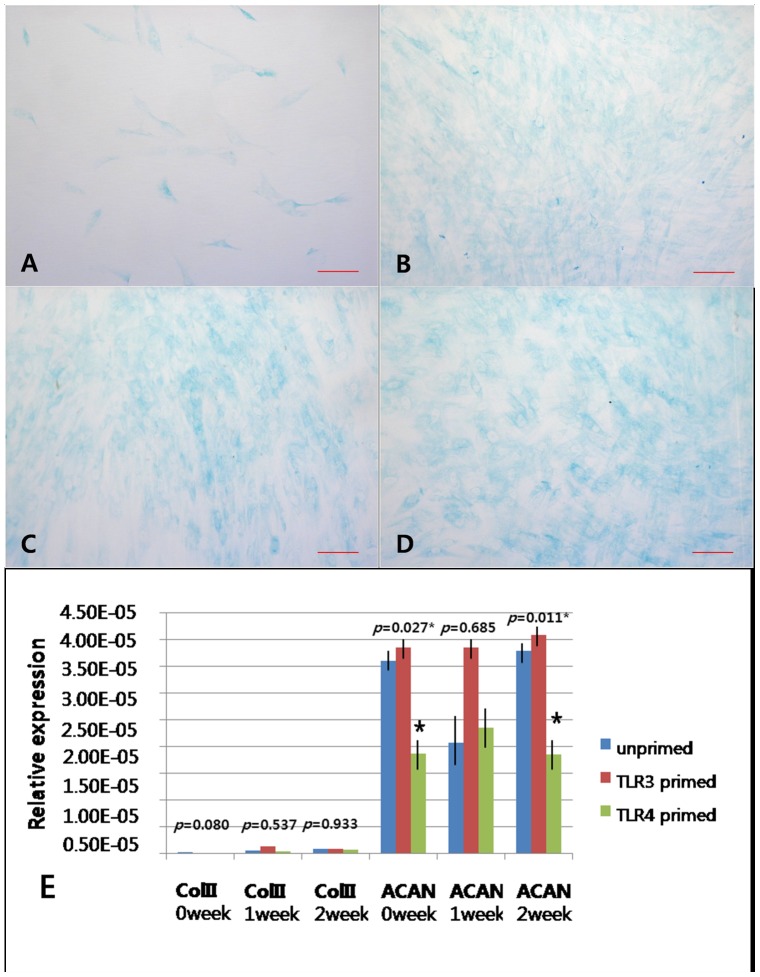
TLR-related change in the chondrogenic differentiation potential of hTMSCs. Cells were cultured in chondrogenic induction medium (A). Cells demonstrated a sulphated extracellular matrix with toluidine blue staining over the 2-week period (B–D). B–D: toluidine blue staining on cultured hTMSCs for all groups. (B: unprimed, C: TLR3 primed, D: TLR4 primed). Magnification: ×400. Scale bars (A–D): 20 µm. No differences in visible color density were observed among the three groups. RT-PCR analysis of type II collagen and aggrecan mRNA expression in hTMSCs during 2 weeks of culture was performed. The expression levels of type II collagen did not differ among the three groups, but aggrecan expression was higher in the unexposed and TLR3 agonist exposure groups than in the TLR4 agonist exposure group.

To verify chondrogenesis, cells exposed to chondrogenic medium displayed the sulphated extracellular matrix of toluidine blue staining unlike cells in the undifferentiated state. However, there were no round shaped chondrocytes like cells and obvious cartilage lacuna. The data from a visual assessment demonstrated that hTMSCs from all groups displayed similar amounts of toluidine blue staining (Figures 7A, 7B, 7C and 7D). mRNA expression of the chondrogenic differentiation markers type II collagen and aggrecan was detected by RT-PCR. The expression levels of type II collagen did not differ among the three groups, but aggrecan expression was higher in the unexposed and TLR3 agonist exposure group than in the TLR4 agonist exposure group (Figure 7E). However, the cells did not exhibited consistently strong and increasing patterns of chondrogenic differentiation markers. Based on these results, hTMSCs did not exihibit consistently strong and increasing expression of chondrogenic differentiation markers regardless of the exposure to TLR agonists.

**Figure 8:**
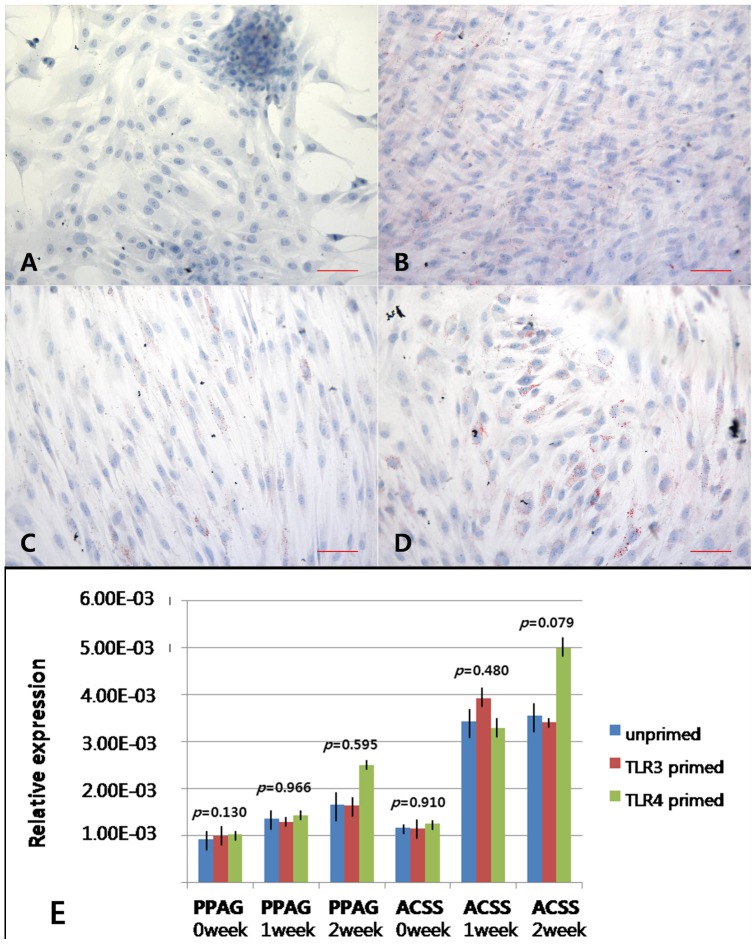
TLR-related changes in the adipogenic differentiation potential of hTMSCs. Cells were cultured in adipogenic induction medium (A). Adipogenesis was detected by the staining of intracytoplasmic lipid droplets with Oil Red O after 2 weeks of culture. (B–D). (B: unprimed, C: TLR3 primed, D: TLR4 primed). Magnification: ×400. Scale bars (A–D): 20 µm. No differences in visible color density were observed among the three groups. RT-PCR analysis of peroxisome proliferator activated receptor r (PPARr) and AcylCoA synthetase (ACS) mRNA expression in hTMSCs was conducted during the 2 weeks of culture. The expression levels of adipocyte-specific genes did not differ among the groups.

After a 2 week culture in adipogenic differentiation medium, the cells acquired an adipocytic phenotype, as evidenced by Oil Red O staining of multi-sized intracytoplasmic lipid droplets in the induced cells. Exposure to TLR agonists (LPS for TLR4 or poly(I:C) for TLR3) had no significant effects on the visible color density of Oil Red O staining (Figures 8A, 8B, 8C and 8D). The data from a visual assessment demonstrated that hTMSCs from all groups showed similar amounts of Oil Red O staining. Cells subjected to adipogenic induction for 14 days consistently displayed strong and increasing expression of mRNAs encoding peroxisome proliferator activated receptor γ (PPARγ) and AcylCoA synthetase (ACS). The expression levels of adipocyte-specific genes did not differ among the three groups (Figure 8E). Considering hTMSCs showed consistently strong and increasing expression of adipogenic differentiation markers regardless of the exposure to TLR agonists, we concluded that adipogenic differentiation of human MSCs was not affected by TLRs.

## Discussion

MSCs exhibit multiple functions and are considered to be important for prospective cell-based therapy. Understanding the factors and mechanisms regulating MSC differentiation, proliferation, and their immunomodulatory activity is crucial and could facilitate therapeutic use of MSCs [3]. There have been many studies regarding the expression of TLRs on MSCs as well as the modulation of MSC biological functions by TLR activation [9]. To date, ten analogues of TLR have been identified in humans. Among them TLR3 and TLR4 have a consistently high expression in human MSC [6]. Consequently, many studies have reported results regarding the role of TLR3 and TLR4 ligands on the ability of human MSCs to modulate differentiation, proliferation, and immune responses.

There is currently no consensus on whether the activation of TLR ligands can affect MSCs potential or not. Several studies have reported the effects of TLR activation on MSC differentiation with contradictory results [10]. Some of the studies found no effects of TLR activation on human MSC proliferation [2]. In contrast, Hwa Cho et al. reported that TLR activation (poly(I:C) (25 µg/ml) and LPS (10 µg/ml)) of adipose derived-MSCs (AD-MSCs) inhibited their proliferation [11]. In particular, inconsistent results have been reported regarding the role of TLR ligands on MSCs' capacity to modulate immune responses. Some groups found that TLR activation (poly(I:C) (30 µg/ml) and LPS (10 µg/ml)) had no significant effect on human MSCs from BM, AD, and Wharton jelly's [2], [12]. However, other groups have reported that TLR activation (poly(I:C) (50 µg/ml) and LPS (5 µg/ml)) may modulate the immunosuppressive properties of human BM-MSCs, although in very different ways [13]. Recently, Waterman et al. proposed that MSCs does not simply possess the immunosuppressant effect but can polarize to a pro-inflammatory (TLR4-primed) or an immunosuppressive (TLR3-primed) phenotype through TLR signaling (poly(I:C) (1 µg/mL) and LPS (10 ng/mL)). The authors suggested that the polarizing effects of TLR priming may also explain the contradictory results obtained so far on the effects of TLRs on immunomodulation by MSCs [10].

MSC niches can exist in each tissue with functional differences [5]. Human MSC would therefore exhibit differences that affect their functional properties according to the source from which they are derived [6]. The respiratory tracts are continuously exposed to external environments such as antigens, allergens, and physical stimuli [7], which can make the characteristics of hTMSCs different from those of inner tissue derived MSCs such as BM and adipose tissue. In previous studies, hTMSCs had approximately thirty times higher yield than AD and BM-MSCs and exhibited approximately five times higher proliferation than BM-MSCs. In addition, it was shown that the donor age and cell passage number does not affect the characterization of hTMSCs in contrast with other human MSCs [14], [15]. Therefore, we evaluated whether hTMSCs would express high levels of TLR3 and TLR4 compared with TLR2 and TLR5. We also evaluated whether the exposure of TLR agonists such as LPS and poly(I:C) could induce cytokine and chemokine production by these cells while influencing their proliferation and differentiation potential toward osteoblasts, chondrocytes, and adipocytes.

The TLR profile of hTMSCs is not yet described. We observed by flow cytometry that these cells express TLR2, TLR3, TLR4, and TLR5, with particularly high expression of TLR3 and TLR4. These findings are slightly different from the results recently reported by Hwa Cho et al., which showed high expression of TLR2, TLR3, and TLR4 at the mRNA level in human AD-MSCs [11]. However, our results are in agreement with Liotta et al.'s showing that TLR3 and TLR4 mRNAs were highly expressed by MSCs in BM-MSCs [3]. These partial discrepancies could be due to differences in the tissue which the MSCs were derived from as well as culture conditions. In addition, the degree of the expression of TLR3 and TLR4 in hTMSC was relatively weak compared to those in AD and BM-MSCs. It is known that despite high TLRs expression in MSCs from different sources at the mRNA level, the TLRs expression at the protein level seems to be low and is often difficult to detect by flow cytometry [2]. This could explain the relatively low expression of TLRs by flow cytometry in our study compared to high expression by RT-PCR in other studies.

To determine whether hTMSCs are capable of immunomodulation, secretion of cytokines and chemokines and expression of TLRs in hTMSCs after the exposure of TLR agonists were analyzed. hTMSCs constitutively secrete high amounts of IL-6 and IL-8, but not IL-10, TNF-α, or IFN-γ. These results showed a similar pattern of inflammatory mediator secretion with previous studies of nasal mucosal MSCs [16]. Regarding the responsiveness of MSCs to TLRs agonists, Tomchuck et al. reported that, following stimulation of TLRs with low-level (the same concentration of LPS and Poly (I:C) with our study) and long-term (48 hours) TLR-priming protocol, the secretion pattern of TLR3-primed secretion patterns on the BM-MSCs appeared to favor IL-10 and IL-12, whereas TLR4-primed cells tended to favor IL-1β, IL-6, and TNF-α [17]. Waterman et al. showed that TLR3-primed effect on BM-MSCs secretion of IL-4, IL-10, IP-10, and RANTES and TLR4 signaling was upstream of IL-6 and IL-8 by low-level and short-term TLR-priming protocol (the same protocol with our study) [8]. In our study, the only TLR4 agonist LPS significantly induced expression of IL-6, IL-8, GM-CSF, IL-12, TNF-α, IP-10, and RANTES in hTMSCs and TLR3 agonist poly (I:C) had no significant effect on both secretion of these cytokines. However, despite statistical significance, the upregulation of cytokines such as IL-12 and TNFα would be doubtful because the levels of these cytokines were very low (approximately 1 pg/ml). These results showed the contradiction to the polarizing process of Waterman et al showing that MSCs can polarize to a pro-inflammatory (TLR4-primed) or an immunosuppressive (TLR3-primed) through TLR signaling [8]. Additionally, TLR3 agonist poly (I:C) had no significant effect on expression levels of TLR2, TLR3, TLR4 and TLR5, as measured by flow cytometry but LPS significantly up-regulated the expression of TLR2 and TLR4.

Our results are reminescent of those recently described by Raicevic et al. in studying MSCs from umbilical cord Wharton's Jelly. Even though the cells expressed TLR, they did not release TLR-inducible cytokines (poly(I:C) (30 µg/mL) and LPS (10 µg/mL)), implicating an ineffective downstream signaling as the mechanism involved in the inflammatory anergy [18]. Although hTMSCs shared a similar pattern of TLR expression with BM-MSC, the responsiveness of TLR3 expressed by hTMSCs were inactive upon TLR ligation (poly(I:C) (10 µg/ml) and LPS (100 ng/mL)) as shown by the constancy of cytokine release, a behavior unlike that of BM-MSCs [3]. These discrepancies would result from a short incubation with TLR agonist in comparison with other studies. However, Liotta et al. reported that poly(I:C) and LPS induced NF-κB translocation to the nucleus of BM-MSCs within 30 minutes after incubation of TLR-agonists and TLR activation in BM-MSCs also led to the secretion of a variety of cytokines and chemokines. Based on these results, they suggested that TLRs expressed by BM-MSCs were functional. [3] Additionally, Waterman et al. showed the short-term TLR-priming of BM-MSCs secreted various inflammatory mediators. Therefore, our results could be explained by Raicevic et al's suggestion that MSCs' resistance to the effects of TLR ligation are due to two main mechanisms: (a) lack of expression of some TLRs and (b) expression of non-functional TLRs [18]. We theorized that hTMSCs would express non-functional TLR3s because their ligation did not trigger the secretion of any of the cytokines tested. However, it would be needed to verify whether there would be the translocation of NF-κB in hTMSCs by TLR3-priming for acknowledging the mechanism of TLR3 activation on the immunomodulation of hTMSCs

To determine whether TLRs are involved in tri-lineage (cartilage, bone, fat) differentiation of hTMSCs, the cells were treated with LPS and poly(I:C) and cultured in standard inductive medium. During osteogenic and adipogenic differentiation of hTMSCs, none of the agonists tested increased the intensity of the histologic staining. The expression levels of the osteoblast and adipocyte-specific genes (Runx2, BMP-2, type I collagen, PPARγ, and ACS) displayed a consistently strong and increasing pattern regardless of the exposure of TLR agonists. These results indicate that TLR signaling does not promote osteogenic and adipogenic differentiation in hTMSCs.

hTMSCs exposed to chondrogenic medium displayed a weak sulphated extracellular matrix and no round shaped chondrocyte-like cells with toluidine blue staining during all differentiation periods. Although TLR4 agonist exposure significantly down-regulated the expression levels of aggrecan and not type II collagen, the expression levels of chondrocyte-specific genes did not display a consistent and steadily increasing pattern. These results indicate that all the cells in this study exposed to chondrogenic medium were incapable of differentiating chondrogenic phenotype. In contrast to our previous study showing that hTMSCs differentiate easily into chondrocytes under in vitro culture, the expression of markers of chondrogenic differentiation in this study was very low. This may be due to differences in the characteristics of the hTMSCs cultured from different donors as well as culture conditions, since differences in experimental settings and culture methods may lead MSCs to behave differently [19]. Future studies will evaluate the chondrogenic differentiation potential of hTMSCs after exposure to TLR agonists.

Most studies have not identified any effects of TLR activation on human MSC proliferation. Only Hwa Cho et al. [11] reported that TLR9 activation of AD-MSCs inhibited their proliferation while the other TLR ligands such as TLR2, TLR3, and TLR5 had no effect. Interestingly, the use of TLR-deficient mouse BM-MSCs provided some insight on the role of TLRs on proliferation as TLR4-deficient BM-MSCs had higher proliferation rates whereas TLR2-deficient BM-MSCs had reduced proliferation compared to wild- type MSCs. In addition, TLR2 and TLR4 activation (LPS (1 µg/mL)) promoted proliferation of mouse BM-MSCs [2]. In our study, during the logarithmic growth period, hTMSCs exposed to the TLR4 agonist expanded more rapidly than those from unprimed and TLR3 primed cells. No significant effect of TLR4 ligands on proliferation in the lag phase would be explained by the fact that cells achieved confluence after a period of 7 days because of the high proliferation rate of hTMSCs. These findings support the hypothesis that TLR activation may affect the proliferating ability of existing or transplanted hTMSCs in vivo.

Our study is the first to demonstrate that the stimulation via TLR4 agonist within hTMSCs leads to the activation of the immune-modulating functions established for MSCs. Interestingly, these activations mediate the secretion of discrete patterns of cytokines and chemokines depending on the TLR ligand used. We also show that TLR4 stimulation particularly promotes the proliferation capabilities of hTMSCs. Therefore, TLR4 stimulation may be one mechanism that specifically drives the proliferation and immune modulating functions of the hTMSCs at injured or stressed sites. hTMSCs may represent a currently underestimated player in local immune regulation with immunological interactions such as the expression of different cytokines or chemokines as well as the response of hTMSCs to selective TLR stimuation. Collectively, our experiments suggest that the upper airway contains a population of MSC with immune modulating functions and these results support recent evidence that MSC play a role in modulating local immunity [20]. However, unlike the polarizing process of Waterman et al according to TLR signaling [8], TLR4 signaling in hTMSCs may be upstream of not only the pro-inflammatory (IL6 and IL8) but also the immunosuppressive mediators (IL-12, CCL10 (IP-10), CCL5 (RANTES)) [11].

Although we could not verify the different mechanisms of TLR stimulation in hTMSCs, these discrepancies are most likely due to the fact that the respiratory mucosa is continuously exposed to enormous amounts of antigens, which makes it necessary for the mucosal immune system to balance pro-inflammatory responses with anti-inflammatory responses [7]. The present work provides evidence that hTMSCs play a pivotal role in both initiating the clearance of pathogens and promoting the repair of injured tissue, raising the possibility that hTMSCs could be employed clinically to augment host defense [21]. In addition, the source from which MSC are derived is of importance for the design of MSC based immunointervention [6]. The accumulation of such data will help to shed more light and clarity on discrepancies in this field.

## Conclusions

These data suggest that hTMSC express relatively high basal levels of TLR3 and TLR4. However, unlike BM and AD-MSCs, the response of hTMSCs to only TLR4 stimulation is related to the immunologic modulation and proliferation of these cells rather than to differentiation of the hTMSCs. These results suggest that understanding the immunomodulatory behavior of MSCs derived from different origins is essential and that hTMSCs may play a central role in local immune regulation of respiratory mucosa. However, additional studies need to be performed to evaluate the chondrogenic differentiation potential of hTMSCs after exposure to TLR agonists. Further evaluation of the immunomodulating properties of hTMSCs would therefore provide a novel target to exploit in the improvement of stem cell-based therapeutic strategies.
